# Effect of micro-algae S*chizochytrium* sp. supplementation in plant diet on reproduction of female rainbow trout (*Oncorhynchus mykiss*): maternal programming impact of progeny

**DOI:** 10.1186/s40104-022-00680-9

**Published:** 2022-03-10

**Authors:** Emilie Cardona, Emilien Segret, Yoann Cachelou, Thibaut Vanderesse, Laurence Larroquet, Alexandre Hermann, Anne Surget, Geneviève Corraze, Frederic Cachelou, Julien Bobe, Sandrine Skiba-Cassy

**Affiliations:** 1 INRAE, Univ. Pau & Pays Adour, E2S UPPA, NuMéA, F-64310, Saint Pée-sur-Nivelle, France; 2 INRAE, UR1037 Fish Physiology and Genomic laboratory, F-35000 Rennes, France; 3 Viviers de Sarrance, F-64490, Sarrance, France; 4 Viviers de Rébénacq, F-64260, Rébénacq, France

**Keywords:** Egg quality, Micro-algae, Nutritional programming, Plant diet, Rainbow trout, Reproduction

## Abstract

**Background:**

The broodstock diet, and in particular the lipid and fatty acid composition of the diet, is known to play a key role in reproductive efficiency and survival of the progeny in fish. A major problem when replacing both fish meal and fish oil by plant sources is the lack of n-3 long chain polyunsaturated fatty acids, such as eicosapentaenoic acid (EPA) and docosahexaenoic acid (DHA). To address this problem, we studied the effect of the plant-based diet supplemented with *Schizochytrium* sp. microalgae, source of DHA, compared to a conventional commercial diet rich in fish meal and fish oil on reproductive performance and egg quality and the consequences on progeny, in female rainbow trout broodstock.

**Results:**

The results demonstrated that DHA-rich microalgae supplementation in a plant-based diet allowed for the maintenance of reproductive performance and egg quality comparable to a conventional commercial feed rich in fish meal and fish oil and led to an increased significant fry survival after resorption. Moreover, when females were fed a plant-based diet supplemented with micro-algae, the 4-month-old progenies showed a significant higher growth when they were challenged with a similar diet as broodstock during 1 month. We provide evidence for metabolic programming in which the maternal dietary induced significant protracted effects on lipid metabolism of progeny.

**Conclusions:**

The present study demonstrates that supplementation of a plant-based diet with DHA-rich microalgae can be an effective alternative to fish meal and fish oil in rainbow trout broodstock aquafeed.

**Supplementary Information:**

The online version contains supplementary material available at 10.1186/s40104-022-00680-9.

## Introduction

Due to the increasing demand for fish and shellfish, aquaculture is playing an increasingly important role in providing an important source of protein for people. In the last 30 years, the increase in aquaculture production has forced a change in fish feed composition, whereby fishmeal (FM) and fish oil (FO) have been increasingly replaced by more available plant sources. Between 1990 and 2018, for example, FM and FO inclusion rates in the diets of Atlantic salmon in Norway declined from 65% to 13% and 24% to 10%, respectively [1]. Despite the fact that FM and FO inclusion rates in aquafeeds have shown a clear downward trend over the last decades [2], their total replacement by plant sources is still not optimal as it leads to reduced feed efficiency, growth and reproductive performances [3, 4].

Carnivorous fish species, such as salmonids, are among the highest consumers of FO and FM [5]. Among salmonids, rainbow trout (*Oncorhynchus mykiss*) farming represented a world production of 811 thousand tons in 2017 [2]. It is the main freshwater production in Europe (over 150,000 t produced by 14 countries in 2017) and intense research efforts are currently focused on replacing marine ingredients (FM and FO) with more readily available plant-based ingredients in their diets [6–11].

In the broodstock diet, especially for rainbow trout, the problem of FM and FO replacement is accentuated. For successful reproduction, the protein and lipid requirements for broodstock are important and highly specific. Several studies revealed that the replacement of FO and/or FM with plant products in broodstock diet alters broodstock reproductive performances in rainbow trout [3, 12] and in other fish species [13, 14]. Two long-term studies (3 years) evaluated the effect of replacing only FM or both FO and FM with plant products on the reproductive performance of female rainbow trout [3, 12]. Lee et al. [12] investigated the long-term effects of replacement of FM by dietary cottonseed meal on growth and reproductive performance of rainbow trout and showed an important decrease in the survival of individuals at the eyed-stage. While Lazzarotto et al. [3] showed that, despite total replacement of FO and FM by plant products, rainbow trout can successfully produce ova in which neo-synthesized long chain polyunsaturated fatty acids (LC-PUFAs) accumulate, the reproductive performance and egg quality remain negatively impacted by these total replacements notably by reducing egg size and survival of the offspring. A major issue when replacing both FM and FO by plant sources is the lack of n-3 LC-PUFAs, such as eicosapentaenoic acid (EPA) and docosahexaenoic acid (DHA). These fatty acids are important in the fish life-cycle, most notably for their roles in reproduction, egg quality and offspring development [15, 16]. One of the main challenges facing researchers today is to find a solution to this lack of essential fatty acids in the plant-based diet, particularly for broodstock.

Microalgae (i.e. single-celled algae or phytoplankton) are considered a promising alternative as replacements for FM and FO, and also help to ensure sustainability standards in aquaculture. Known as sources of protein, lipid, vitamins, minerals or pigments, microalgae may also be a source of n-3 LC-PUFAs [17]. The marine microalgae *Schizochytrium* sp.*,* for example, contains between 18% and 22% DHA of their dry matter [18]. Supplementation of aquafeed with this microalgae improved the levels of DHA and total n-3 LC-PUFAs in the fillet tissue in channel catfish [19] and in Nile Tilapia [20] but until now, this supplementation has yet to be investigated within rainbow trout broodstock.

Moreover, changes in the diet of the broodstock are not without consequences for the offspring. Many authors have shown the importance of the maternal environment on the development of the offspring in vertebrates [21], including fish [13, 22]. The maternal environment, including nutrition, can influence embryonic and fetal life and thus modify the trajectory of the offspring’s development [23]. In aquaculture, the phenomenon of nutritional programming was previously tested to improve the acceptance of a plant-based diet in rainbow trout [13, 14, 24]. Currently, research on nutritional programming is primarily focused on programming juveniles through early nutritional stimuli, at first feeding [25–32], but research on the maternal role in progeny programming is still in its infancy [13, 14, 33]. Two studies on gilthead seabream [13, 14] demonstrated that it is possible to nutritionally program offspring by replacing fish oil with vegetable oils in the broodstock diet, resulting in an improvement in their ability to grow fast when fed low fish meal and fish oil diets during the grow-out phase.

In the present study, we compared the effects of a plant-based diet supplemented with *Schizochytrium* sp. micro-algae with those of a commercial diet, on reproductive performances and egg quality of female rainbow trout (*Oncorhynchus mykiss*). We also studied the consequences of maternal nutritional history on offspring to assess the implementation of nutritional programming.

## Material and methods

### Ethical statements

Broodstock experimentation was conducted in the Viviers de Rébénacq commercial fish farm (Rébénacq, France) and egg incubation and fingerling rearing were conducted in the Viviers de Sarrance commercial fish farm (Sarrance, France). All fish were reared and handled in strict accordance with French and European policies and guidelines. Fish were monitored daily during the experiment. If any clinical symptoms (i.e. morphological abnormalities, restlessness or uncoordinated movements) were observed, fish were sedated by immersion in MS-222 solution at a concentration of 50 mg/L and then euthanized by immersion in a MS-222 solution at a concentration of 400 mg/L (anesthetic overdose) for 5 min.

### Broodstock diet and challenge diet of progeny

The broodstock experiment was conducted with two different diets: a commercial broodstock diet (Neo repro, Le Gouessant, Lamballe, Côte d’Armor, Brittany, France) containing a mix of FM, FO and plant ingredients (CB), and a plant-based diet completely devoid of FM and FO but supplemented with *Schizochytrium* sp. microalgae biomass (MAB).

The plant-based diet supplemented with microalgae biomass was formulated by INRAE to fulfill the nutritional requirements of rainbow trout [34]. The two diets were produced by the feed company “Le Gouessant” (Lamballe, Côte d’Armor, Brittany, France). Ingredients of the MAB diet are presented in Table 1.

**Table 1:** Ingredients (only for MAB diet) and composition of female broodstock diets

Ingredients, %	MAB	CB
*Schizochytrium* sp. microalgae biomass	6.9	
Corn gluten	19.7	
Soybean meal	14.5	
Pea protein concentrate (Lysamine)	15.0	
Soy protein concentrate	9.6	
Bean protein concentrate	9.0	
Guar 70 PFR Roasted	9.0	
Alfalfa protein concentrate	5.0	
Rapeseed oil	3.2	
Linseed oil	1.1	
Choline chloride 60%	0.3	
Soy lecithin	1.0	
Minerals premix	1.6	
Vitamin premix	1.5	
Dicalcium phosphate	1.2	
L-Lysine	0.8	
L-Méthionine	0.5	
Carophyll pink (astaxanthin)	0.04	
**Proximate composition**		
Dry Matter, %	91.7	92.6
Proteins, % DM	54.1	49.5
Lipids, % DM	12.1	15.2
Carbohydrate, % DM	11.5	15.3
Ash, % DM	5.2	12.2
Energy, kJ/g DM	23.3	22.2
Cholesterol, mg/100 mg of DM	24.9	177.6
Phytosterol, mg/100 mg of DM	180.5	53.7

*DM* = dry matter.

The diet used to challenge the progeny was similar to the plant-based diet supplemented with *Schizochytrium* sp. microalgae used for broodstock (vegetable diet) but modified to respond to the specific needs of juveniles (MAO diet). Micro-algae biomass was replaced by micro-algae oil due to a supply problem concerning micro-algae biomass during the production of the experimental diet (Table 2). The ingredients list of the commercial diet (CO, Neo supra, Le Gouessant, Lamballe, Côte d’Armor, Brittany, France) fed to progeny cannot be published because of the confidentiality agreement with the feed manufacturer but the proximate composition is available (Table 2). The fatty acid profile for the progeny diets is presented in Additional file 1: Table S1.

**Table 2:** Ingredients (only for MAO diet) and composition of progeny diets

Ingredients, %	MAO	CO
*Schizochytrium* sp. microalgae oil	2.6	
Corn gluten	18.0	
Soybean meal	5.1	
Pea protein concentrate	20.5	
Soy protein concentrate	23.6	
Bean protein concentrate	4.0	
Guar 70 PFR Roasted	2.0	
Alfalfa protein concentrate	5.0	
Rapeseed oil	9.2	
Linseed oil	2.0	
Choline chloride 60%	0.3	
Soy lecithin	2.5	
Minerals premix	1.5	
Vitamin premix	1.2	
Dicalcium phosphate	1.2	
L-Lysine	0.6	
L-Methionine	0.6	
**Proximate composition**		
Dry Matter, %	97.2	92.1
Proteins, % DM	56.9	52.0
Lipids, % DM	23.3	17.3
Ash, % DM	6.0	10.4
Energy, kJ/g DM	25.7	19.2
Cholesterol, mg/100 mg of DM	38.6	301.1
Phytosterol, mg/100 mg of DM	268.0	5.4

*DM* = dry matter.

### Broodstock breeding and experimental design

Before any manipulation, fish were fasted for 24 h and anesthetized with MS-222 (50 mg/L). Female rainbow trout from an autumn-spawning strain were held under natural photoperiod until their first reproduction (2 years) in the Viviers de Rebenacq fish farm. After the first spawning, each fish was individually weighed and identified with a RFID Pit-tag (Biolog-id, Bernay, France). A total of 180 females, with a biomass of 25 kg/m^3^ (initial weight: 2227 ± 268 g), were then randomly transferred into 4 outdoor tanks with a volume ranging from 3.5 to 4.5 m^3^ (*n* = 90 females per treatment). Each diet was assigned to two tanks. Females were fed the experimental diets after the first reproduction and continued until the third reproduction.

The feeding rate used in the experimental design was established according to a conventional feeding table provided by the feed manufacturer (Le Gouessant, Lamballe, Côte d’Armor, Brittany, France). This table was calculated according to fish weight and water temperature.

Two reproductive cycles were followed during this trial. For more fluidity, the cycle between the first and the second reproduction period and between the second and the third reproduction period will be referred to as Cycle 2 (from week 0 to week 39) and Cycle 3 (from week 40 to week 80), respectively. It should be noted that after the spawning period of Cycle 2, some fish were randomly eliminated to ensure that the second cycle started with an initial fish biomass of 25 kg/m^3^.

During these two nine-month cycles, an artificial photoperiod regime was applied to trigger a second reproduction during summer, and a third reproduction during spring. The water renewal rate was 200% per hour. Fish are reared in outdoor tank with a water turnover of 200%. Temperature was checked twice a day to adjust feeding rate. During experiment, temperature fluctuated with the season (from 8 °C to 13 °C). In order to monitor individual growth and to adjust the distribution of feed, fish were individually weighed every 6 weeks during Cycle 2 and at different stages of the reproductive cycle during Cycle 3 (see paragraph Sampling for more details). During Cycle 3, at the same time as the fish were weighed, blood samplings were performed after fish were sedated with MS-222 (50 mg/L).

**Fig. 1:**
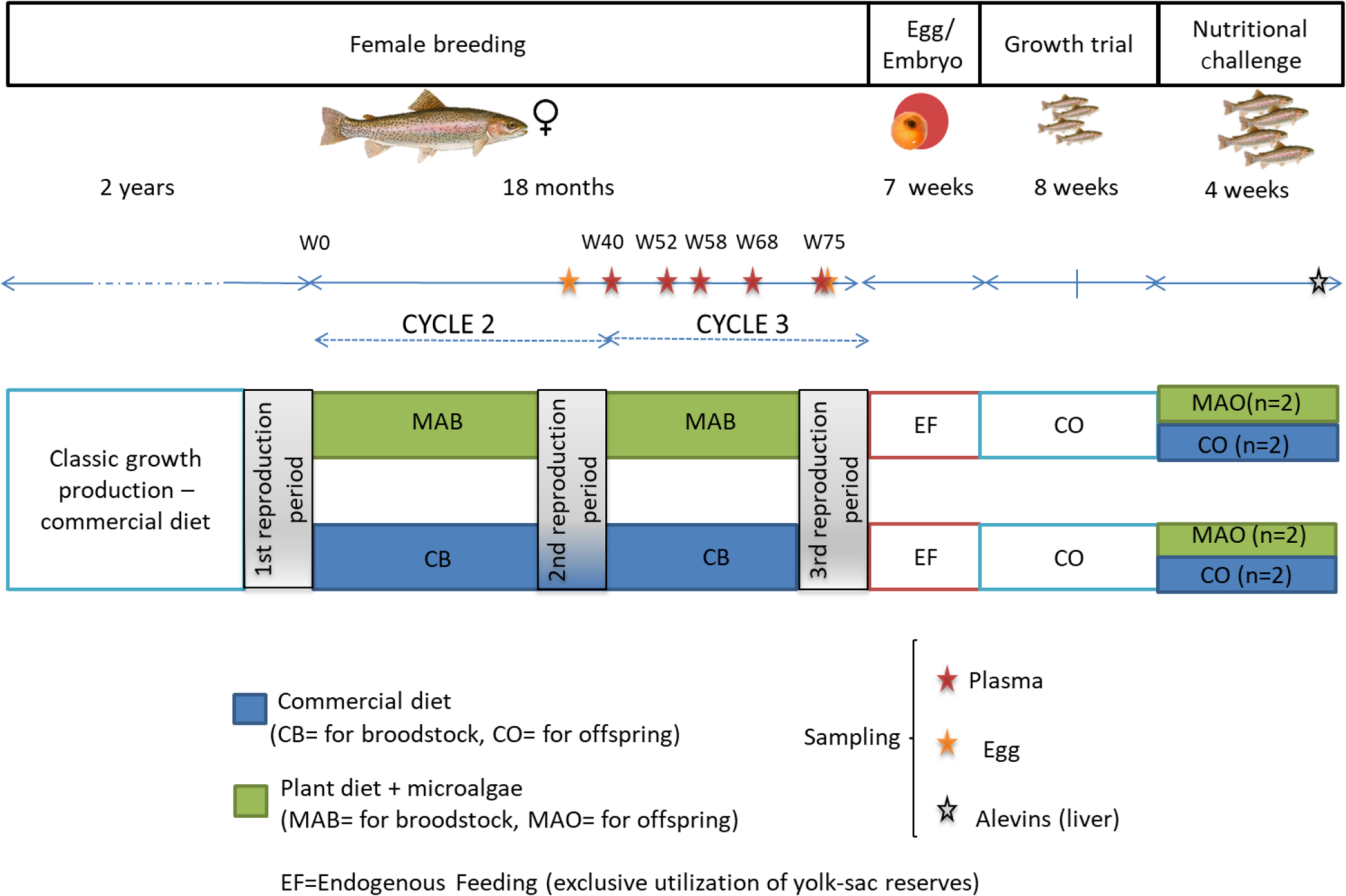
Experimental design (W=Week of rearing)

The experimental design is summarized in Fig. 1.

### Reproduction, fertilization and incubation

During the spawning season, females were anesthetized with MS-222 (50 mg/L) and checked for ovulation once a week by applying manual pressure on the abdomen of the fish. Feeding was stopped for all animals when the first ovulation was detected. Ovulating females were weighed and manually stripped. The ovarian fluid and eggs produced by each female were weighed and eggs were sampled during the second and third reproduction periods.

During Cycle 3, egg batches from 10 females by treatment randomly selected at the peak of spawning were transferred to Viviers de Sarrance facilities. Approximately 400 eggs from each batch were individually fertilized with a pool of sperm collected from neo-males from the fish farm that were fed a commercial diet ad libitum. Three microliters of a pool of semen obtained from three neo-males presenting the highest sperm motility were pre-diluted in storFish solution (IMV technologies, L’Aigle, France) and added to each group of eggs. Fifteen mL of Actifish solution (sperm motility activating saline solution, IMV technologies, L’Aigle, France) were added to the eggs. Five min later, the sperm motility activating solution was drained and egg batches were transferred into individual incubators in recirculated water units. The temperature of the water remained constant (9 °C) throughout the incubation period. Dead eggs and embryos were periodically manually counted and removed. Survival at completion of yolk-sac resorption were monitored and calculated as a percentage of the initial number of eggs used for the fertilization. The occurrence of noticeable morphological malformations (spinal cord torsion, head or caudal fin malformations, etc.) at yolk-sac resorption was also recorded.

### Growth trial and nutritional challenge on progeny

After resorption of the yolk vesicle, pools of fry were generated according to the maternal nutritional history. Then, fingerlings were transferred in racks with well-aerated water at 16 ± 2 °C, under natural photoperiod. During this growth period, duplicate groups were manually fed a commercial diet (Le Gouessant, neo supra, Lamballe, Côte d’Armor, Brittany, France), fish were fed 4 times per day until first feed refusal (*n* = 2 racks per treatment). After 4 weeks of trial, groups were divided into two to decrease density and kept for the following trial (*n* = 4 ranks per treatment). The feed grain size was adapted to the size of the fry. Biomass of each tank was weighed every 2 weeks to monitor growth. After 4 and 8 weeks of trial, fish were counted and weighed to calculate percent survival (*n* = 2 at week 4; *n* = 4 at week 8). After 8 weeks of trial, fish were weighed to calculate individual body weights (*n* = 30).

After 8 weeks of trial, all groups were adjusted to include 150 fingerlings per rack. To determine the effect of the maternal nutritional history on the ability of the progeny to use a plant-based diet, half of the fish were challenged with the MAO diet for a period of 4 weeks whereas the other half continued to be fed the commercial diet (CO). Fish were manually fed 4 times per day. At the end of nutritional challenge, all fish were counted to calculate percent survival rate and 30 fish per condition were weighed individually.

The goal of this study was to compare the maternal effect on progeny, and the effect of the diet supplied to the offspring will not be discussed here.

### Sampling

#### Female broodstock

During Cycle 3 of the experiment (week 40 to week 80), blood sampling was performed at different times (Fig. 1): at week 40 (just after the reproduction period of Cycle 2), 52 (before the change of photoperiod, i.e. early vitellogenesis), 58 (i.e. mid vitellogenesis), 68 (i.e. last step of vitellogenesis) and during spawning season (week 75). Blood samples were collected from the caudal vein (approximatively 4 mL per fish) using EDTA-treated syringes (sodium EDTA, 10%). Blood samples were centrifuged (3500 × *g*, 15 min) and plasma was collected, frozen in liquid nitrogen and stored at − 80 °C until further analysis. Eggs (approximatively 30 g per fish) were sampled during spawning for each reproductive cycle and frozen at − 20 °C until further biochemical analysis.

#### Progeny

At the end of the nutritional challenge (12 weeks), sampled fish were sedated by immersion in an iso-eugenol solution at a concentration of 20 mg/L and euthanized by immersion in an iso-eugenol solution at a concentration of 100 mg/L (anesthetic overdose) for 3 min. Liver (ten fish per condition) were dissected, immediately frozen in liquid nitrogen and stored at − 80 °C until further molecular analysis. Twenty fish were sampled for proximal composition and fatty acid profile analysis, and stored at − 20 °C until further analysis.

### Plasma cholesterol analysis

Cholesterol levels were measured from the plasma of females whose eggs were used to produce fingerlings for the nutritional challenge (*n* = 10 per treatment) during Cycle 3. Plasma cholesterol level was determined using commercial kits adapted to a microplate format, according to manufacturer recommendations (Sobioda, Montbonnot-Saint-Martin, France).

### Automatic egg size, number and integrity analysis

Stripped unfertilized eggs (approximatively 400 eggs, about 25 to 30 g) were placed in 150 mL containers and water was added to hydrate the eggs. After 24 h of hydration, a picture of the sampled eggs was obtained using the VisEGG shooting system consisting of a light tablet and digital SLR camera (canon EOS 1000D, resolution: 10.1 M pixels) equipment, as previously described [35]. Pictures were analyzed with Visilog 7.3 software (Thermo Scientific) allowing for automatic measurements to be made (number and size of the eggs and white egg percentage). The total number of ovulated eggs per female could be back calculated using the number of eggs measured and the total weight of the egg batch analyzed. The presence of white egg was interpreted as an indirect measure of egg integrity, the “whitening” being the result of water entry into the egg and through the vitelline membrane [36]. These white eggs were considered non-viable due to their lack of physical integrity.

The total number of ovulated eggs per female correspond to the individual absolute fecundity. Individual fecundity was divided by the weight of the fish to give the value for relative fecundity in number of eggs/kg of female.

### Diet and fish proximate composition analysis

Proximate composition of the diets and biochemical composition of the progenies were determined according to the following procedures: dry matter after drying at 105 °C for 24 h, protein (N × 6.25) by the Kjeldahl method after acid digestion, ash by incineration at 550 °C for 16 h and gross energy in an adiabatic bomb calorimeter. Total lipids were extracted using dichloromethane/methanol (2:1, v/v), containing 0.01% butylated hydroxytoluene (BHT) as an antioxidant, according to Folch et al. [37].

Dietary levels of cholesterol and phytosterol were determined by gas chromatography according to the norm NF EN ISO 12228-1 [38].

### Lipid and fatty acid analysis

Diets and eggs (10 eggs batches per treatment) from each cycle of reproduction were analyzed for lipids and fatty acid composition. Egg samples were of the same origin as the eggs used for monitoring fertilization and incubation. Total lipids were extracted by dichloromethane/methanol (2:1, v/v), containing 0.01% of butylated hydroxytoluene (BHT) as antioxidant, according to Folch et al. [37]. Fatty acid methyl esters (FAME) were prepared by acid-catalyzed transmethylation, using boron trifluoride according to Shantha et al. [39]. Fatty acid methyl esters were analyzed with a Varian 3900 gas chromatograph equipped with a fused silica DB Wax capillary column (30 m × 0.25 mm internal diameter, film thickness 0.25 μm; JW Alltech, France). Injection volume was 1 μL, using helium as carrier gas (1 mL/min). The temperatures of the injector and the flame ionization detector were 260 °C and 250 °C, respectively. The thermal gradient was as follows: 100–180 °C at 8 °C/min, 180–220 °C at 4 °C/min and a constant temperature of 220 °C for 20 min. The fatty acids (FAs) were identified by comparing their retention times with a known standard mixture (Sigma, St Louis, MO, USA) and peaks were integrated using Varian Star Chromatography Software (Star Software, version 5). The results for individual FA were expressed as percentage of total identified FA methyl esters.

### Molecular analysis on progenies

RNA extractions were carried out on individual liver samples for 10 fry per condition. Livers were homogenized in trizol reagent (Ambion) at a ratio of 1 mL for 100 mg of tissue with precyllis®24 (Bertin Technologies) and total RNA was extracted according to manufacturer’s instructions. The quantity and quality of RNA were assessed by measuring their absorbance at 260 and 280 nm using a Nanodrop 1000 Spectrophotometer (Thermo Scientific) associated with ND-1000 V3 7.0 software. Gene expression was assessed as follows: 1 μg of total RNA was subsequently reverse-transcribed to cDNA using the super script RNAse H-reverse transcriptase kit (Invitrogen) with random primers (Promega, Charbonnières, France). Luciferase control RNA (Promega) was added to each sample at the beginning of reverse transcriptase. The levels of messenger RNAs (mRNA) (including paralogs for some of them) were measured in the liver of progenies at the end of the growth period (fed the C diet) and after the 4-week nutritional challenge. Genes subjected to RT-PCR analysis were related to lipogenesis (ATP cytrate lyase (*ACLY*) and fatty acid synthase (*FAS*)), β-oxidation of long-chain fatty acids (carnitine palmitoyl transferase 1 (*CPT1a*), hydroxyacylcoA dehydrogenase (*HADH*) and acylcoA oxidase (*ACOX*)), long-chain fatty acid biosynthesis (acyl-CoA desaturase - delta-9 desaturase (*SCDB)*, elongation of very long chain fatty acids proteins 2 (*ELOVL2b*) and 5 (*ELOVL5a*, *ELOVL5c)* and fatty acid desaturase 2 (*FADS2a, FADS2b* and *FADS2c*)) and cholesterol metabolism (3-hydroxy-3-methylglutaryl-CoA synthase 1 (*HMGCS*), 3-hydroxy-3-methylglutaryl-CoA reductase (*HMGCRa*, *HMGCRb)*, cholesterol 7 alpha hydroxylase a (*CYP7a*) lanosterol 14-alpha demethylase (*CYP51a*, *CYP51b),* ATP-binding cassette sub-family A member 1-like (*ABCA1)*, ATP binding cassette subfamily G member 5 and 8 (*ABCG5*, *ABCG8*) and 7-dehydrocholesterol reductase (*DHCR7a* and *DHCR7b*).

Quantitative RT-PCR analyses were performed with the Roche Lightcycler 480 system (Roche Diagnostics, Neuilly-sur-Seine, France). The reaction mix was 6 μL per sample, including 2 μL of diluted cDNA template (1:25), 0.12 μL of each primer (10 μmol/L), 3 μL of Light Cycler 480 SYBR® Green I Master mix and 0.76 μL of DNAse/RNAse-free water (5 Prime GmbH, Hamburg, Germany). The qPCR protocol was initiated at 95 °C for 10 min for the initial denaturation of the cDNA and hot-start Taq-polymerase activation, followed by 40 cycles of a two-step amplification program (15 s at 95 °C; 10 s at 60 °C). Melting curves were monitored systematically (temperature gradient 0.11 °C/s from 65 to 97 °C) at the end of the last amplification cycle to confirm the specificity of the amplification reaction. Each qPCR assay included replicate samples and negative controls (reverse transcriptase- and cDNA template-free samples). Data were subsequently normalized to the exogenous luciferase transcript abundance. Sequences of primers are presented in Additional file 2: Table S2.

### Statistical analysis

Results are expressed as means and standard deviations. A statistical analysis of the data was carried out using R studio software (version 4.0). A two-way ANOVA was performed to assess the effect of dietary treatment and cycle of reproduction for reproductive performance parameters, egg quality as well as for molecular and biochemical results on eggs and progeny. A one-way ANOVA was performed to assess the maternal origin and diet on progeny individual weight. A Kruskal-Wallis test was carried out to assess the effect of maternal nutritional origin on survival percent of broodstock and on survival percent, evolution of weight means during rearing and proximate composition of progeny.

The adequacy of the final ANOVA results was assessed by making residual plots to check the normality. This analysis did not reveal abnormalities, and thus the analysis was validated. When an interaction was found to be significant, the means for all treatments were compared using a Tukey’s post hoc analysis. Growth monitoring analyses of broodstock and plasma cholesterol analyses were performed on the same animals during different sample times and analyses can be considered as dependent. Thus, in this case, the data followed a normal distribution and a linear mixed model was applied (package LME4 with R studio software). The adequacy of the final linear model was assessed by making residual plots to check the normality. This analysis did not reveal abnormalities, and thus the analysis was validated. As no significant rearing tank effect was observed, we were thus able to only consider feeding procedure as variable factor.

## Results

### Female

#### Biometric parameters of females

**Fig. 2:**
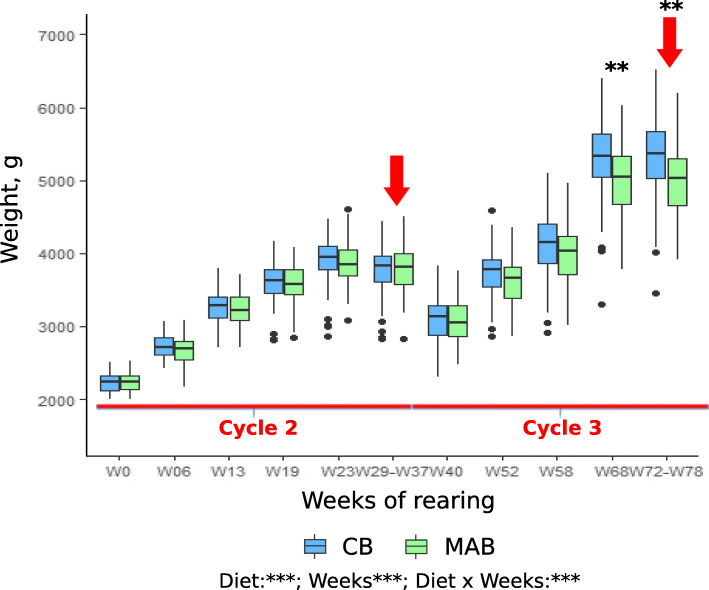
Evolution of female weight according the diet treatment. The red arrows represent the spawning periods. The measurements were carried out on the same females at different times; data can be considered as dependent. Linear mixed model was applied, the best model was then selected using the Akaike Information Criterion (AIC). Replicates correspond to different individual females (*n* = 90 per treatment). “n.s”: not significant; “*”: *P* < 0.05; “**”: *P* < 0.01; “***”: *P* < 0.001

No difference in percent survival was detected between treatments for female broodstock (i.e. percent survival was above 90% for both Cycle 2 and Cycle 3) (see Table 1). The evolution of female weights is presented in Fig. 2. Compared to the CB diet, the MAB diet significantly reduced the growth of females (*n =* 90 females per treatment; *P* < 0.0001). Tukey’s post hoc analysis revealed that the differences are significant at the end of Cycle 3 (from W68). MAB-fed females presented significant lower body weights (− 5.4%) at the end of the trial.

#### Plasma cholesterol analysis

**Fig. 3:**
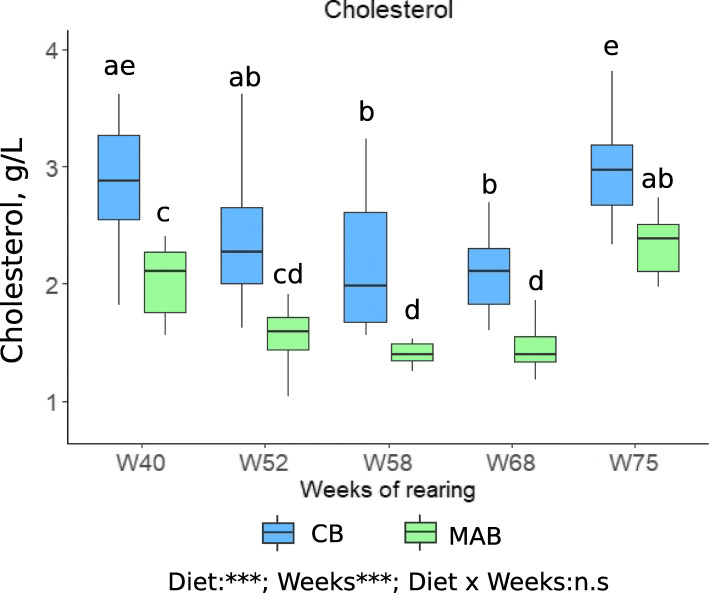
Evolution of plasma cholesterol concentrations over the vitellogenesis during Cycle 3. Plasma sampling was performed at different times during the reproductive Cycle 3: just after the second reproduction period (week 40, W40), before the change of photoperiod, i.e. early vitellogenesis (week 52, W52), at mid vitellogenesis (week 58, W58), at the last step of vitellogenesis (week 68, W68) and during spawning season (week 75, W75). . The measurements were carried out on the same animals (females were individually identified with a RFID Pit-tag) at different times; data can be considered as dependent. Linear mixed model was applied; the best model was then selected using the Akaike Information Criterion (AIC). Replicates correspond to different individual females (*n* = 10 per treatment). “n.s”: not significant; “*”: *P* < 0.05; “**”: *P* < 0.01; “***”: *P* < 0.001. Different letters indicate significant differences between maternal origin, which were investigated with a Tukey post hoc test

The evolution of cholesterol level in plasma during reproductive Cycle 3 (W40 to W68) and spawning period (W75) are presented in Fig. 3. Cholesterol concentration was significantly affected during the week of rearing, i.e. by the stage of vitellogenesis (*n =* 10 females per treatment; *P* < 0.001). The level was relatively high after spawning during Cycle 2, then gradually decreased to W58 and increased again until spawning during Cycle 3. Cholesterol levels are significantly higher in the plasma of CB-fed females throughout the reproductive cycle than in MAB-fed females (*P* < 0.001).

#### Reproductive performance in females, and egg and progeny qualities

Data on reproductive performances as well as egg and fingerlings qualities are reported in Table 3.

**Table 3:** Reproductive performances of females, egg quality and fingerling biometric parameters according to diet treatment during reproductive Cycle 2 and Cycle 3

	Cycle 1		Cycle 2		Statistical results			
Diets	CB	MAB	CB	MAB	Diet		Cycle	Diet × Cycle
**Female biometric parameters and reproductive performances**								
Survival, %	90.32 ± 2.38	91.81 ± 0.71	94.10 ± 3.78	90.00 ± 14.14	n.s		n.s	n.s
Weight female at spawning time, g	3770.39 ± 349.83	3799.18 ± 310.41	5285.21 ± 623.48	5016.32 ± 510.36	n.s		***	**
Egg spawn weight, g	551.91 ± 123.11	542.98 ± 119.53	682.56 ± 135.11	675.02 ± 133.11	n.s		***	n.s
Absolute fecundity, eggs per female	7887.09 ± 2008.14	8086.81 ± 2046.72	9175.20 ± 2005.94	9988.04 ± 2173.13	n.s		***	n.s
Relative fecundity, eggs/kg of female	2108.84 ± 543.57	2134.54 ± 534.79	1764.04 ± 450.98	2007.70 ± 451.19	n.s		***	n.s
**Egg quality**								
Egg diameter, mm	5.20 ± 1.11	5.15 ± 0.19	4.90 ± 0.48	4.64 ± 0.63	**		***	*
Coefficient of variation of egg diameter	3.29 ± 1.11	3.15 ± 0.88	3.07 ± 0.51	3.03 ± 0.43	n.s		n.s	n.s
White eggs, %	6.41 ± 8.58	6.66 ± 9.98	8.89 ± 16.72	2.88 ± 5.13		n.s	n.s	*
**Embryo development**								
Fry survival after resorption, %	_	_	47.58 ± 33.65	70.29 ± 17.55	*		_	_
Fry weigth, mg	_	_	12.99 ± 1.48	12.53 ± 1.52	n.s		_	_
Malformation rate of fry, %	_	_	3.87 ± 5.55	4.30 ± 6.63	n.s		_	_
No resorption rate of fry, %	_	_	0.40 ± 0.82	0.41 ± 0.35	n.s		_	_

Values are means ± s.d. Two-way analysis of variance was carried out in order to assess effects of diet and cycle of reproduction. Replicates to asses statistical effect on reproductive performances of females and egg quality correspond to different individual females (*n* = 90 females per treatment). Replicates to asses statistical effect on fingerling biometric parameters correspond to different individual egg batches (*n* = 10 egg batches per treatment). “n.s”: not significant; “*”: *P* < 0.05; “**”: *P* < 0.01; “***”: *P* < 0.001

At each reproductive cycle, the absolute and relative fecundity as well as the spawn egg weight were similar between the two dietary conditions. In terms of egg quality, a significant interaction between the diet and the reproductive cycle was observed on a percentage of non-viable eggs (i.e. white eggs) and the egg diameter. Therefore, during the reproduction of Cycle 3, MAB diet led to smaller eggs (4.64 mm vs. 4.90 mm, respectively for eggs from MAB-fed females or CB-fed females) but resulted in fewer non-viable eggs (i.e. higher egg integrity; 2.9% vs. 8.9% respectively for eggs from MAB-fed female sand CB-fed females). Diet had no impact on egg size variability.

Fry survival after resorption was significantly higher for eggs originating from MAB-fed females compared to those from CB-fed females (70.3% vs. 47.6% for fry from females fed the MAB diet compared to the CB diet; *n =* 10 eggs batches per treatment *P* = 0.02). No maternal effect was observed on the malformation rate and fry weight at yolk-sac resorption.

#### Lipid composition and fatty acid (FA) profile of broodstock diet and eggs

The proximal composition and the FA profiles of the diets are presented in Table 1 and Table 4, respectively. The CB diet presented a higher level of lipids, including seven times more cholesterol, and a higher level of starch. Inversely, the MAB diet presented a higher level of phytosterols (3.4 more than the CB diet). Dietary FA profiles showed differences between the two diets. One of the largest differences, was attributed to monounsaturated fatty acid (MUFA), specifically the 16:1 fatty acid, that was present in very small quantities in the MAB diet (0.44%) while its proportion reached 9.21% of total FA in the CB diet. Inversely, the 18:1 fatty acid represented 24.4% of total FA in the MAB diet whereas it accounted for only 14.5% of the total fatty acids in the CB diet. The MAB diet also presented 2.5 times more n-6 PUFA. The difference was essentially due to the higher proportion of linoleic acid (18:2 n-6; LA) and docosapentaenoic acid (22:5 n-6; DPA) (2.5 × and 33.9 × higher than CB diet, respectively). In contrast, arachidonic acid (20:4 n-6; ARA) was higher in CB than in the MA diet (4.3 × higher) although its proportion remained lower than 1% of the total FA. The proportion of n-3 PUFA was 3.3 times lower in the MAB diet than in the CB diet. This difference was mainly explained by the lower proportion of eicosapentaenoic acid (20:5 n-3; EPA) since it was 41.9 less concentrated in the MAB diet than in the CB diet. However, higher proportions of α-linolenic acid (18:3 n-3; ALA) and docosahexaenoic acid (22:6 n-3; DHA) (8.2 and 2.8-fold, respectively) were present in the MAB diet.

**Table 4:** Main fatty acids (% of total FA) of broodstock diets (C and MA)

	Broodstock diet	
Saturated fatty acids (SFA)	CB	MAB
14:0	6.30 ± 0.06	0.47 ± 0.04
16:0	18.81 ± 0.12	17.54 ± 0.37
18:0	3.14 ± 0.38	2.39 ± 0.02
Total saturated FA (SFA)	29.27 ± 0.81	21.46 ± 0.11
**Monounsatured fatty acids (MUFA)**		
16:1	9.21 ± 2.65	0.44 ± 0.10
18:1	14.49 ± 0.47	24.08 ± 0.30
20:1	2.14 ± 0.21	0.63 ± 0.07
22:1	1.62 ± 0.05	0.37 ± 0.00
Total monosatured FA	27.59 ± 3.39	25.73 ± 0.04
**Polyunsatured fatty acid (PUFA)**		
16:2 n-4	1.34 ± 0.13	0.01 ± 0.01
16:3 n-4	1.37 ± 0.03	0.08 ± 0.00
16:4 n-1	2.40 ± 0.33	0.04 ± 0.00
**PUFA n-6**		
18:2 n-6	5.74 ± 0.46	20.27 ± 0.96
20:4 n-6	0.78 ± 0.20	0.18 ± 0.16
22:5 n-6	0.14 ± 0.05	4.89 ± 0.15
Total PUFA n-6	9.80 ± 4.20	25.97 ± 0.75
**PUFA n-3**		
18:3 n-3	0.95 ± 0.06	8.76 ± 0.19
18:4 n-3	1.74 ± 0.09	0.11 ± 0.01
20:5 n-3	15.50 ± 0.02	0.37 ± 0.15
22:5 n-3	1.72 ± 0.24	0.08 ± 0.01
22:6 n-3	5.63 ± 1.02	15.50 ± 0.46
Total PUFA n-3	24.29 ± 0.74	16.27 ± 0.60
**Ratio**		
SFA/PUFA	0.73 ± 0.01	0.42 ± 0.01
n3 / n6	3.67 ± 0.31	0.97 ± 0.02
Index Insat	198.83 ± 0.39	216.74 ± 4.61
EPA/DHA	2.80 ± 0.50	0.02 ± 0.01
ARA/EPA	0.05 ± 0.01	0.42 ± 0.26
ARA/DHA	0.14 ± 0.01	0.01 ± 0.01

FA egg profiles were very different between the diets (*n* = 10 eggs batches per treatment) (Table 5). Eggs from MAB-fed females presented a higher proportion of n-6 PUFA (2.3 to 2.8 × more) but a lower proportion of n-3 PUFA (1.3 × more) than eggs from CB-fed females. A higher proportion of 18:1, LA, DPA, ALA, DHA and a lower proportion of 16:1 and EPA were observed in eggs from MAB-fed females compared to eggs from CB-fed females.

**Table 5:** Fatty acid profiles (% of total FA) of eggs from females fed C or MA diet sampled during Cycle 2 and Cycle 3 of the experimentation

	Cycle 2		Cycle 3		Statistical results		
Saturated fatty acids (SFA)	CB	MAB	CB	MAB	Diet	Cycle	Diet x Cycle
12:0	_	_	_	_	_	_	_
14:0	2.27 ± 0.14	0.49 ± 0.03	2.62 ± 0.14	0.45 ± 0.04	***	***	n.s
15:0	0.25 ± 0.01	0.13 ± 0.01	0.26 ± 0.02	0.14 ± 0.01	***	***	*
16:0	15.47 ± 0.50	14.10 ± 0.69	16.14 ± 0.77	15.04 ± 0.40	***	***	n.s
17:0	0.29 ± 0.02	0.17 ± 0.01	0.28 ± 0.03	0.18 ± 0.02	***	n.s	n.s
18:0	5.70 ± 0.38	4.71 ± 0.36	5.91 ± 0.25	5.50 ± 0.35	***	***	**
20:0	0.04 ± 0.01	0.05 ± 0.01	0.05 ± 0.00	0.05 ± 0.01	***	**	n.s
22:0	n.d	n.d	n.d	n.d	_	_	_
Total saturated FA (SFA)	24.03 ± 0.39	19.65 ± 0.99	25.26 ± 0.99	21.35 ± 0.69	***	***	n.s
**Monounsatured fatty acids (MUFA)**							
16:1	5.56 ± 0.23	2.90 ± 0.26	6.12 ± 0.29	2.75 ± 0.32	***	n.s	***
17:1	0.00 ± 0.00	0.00 ± 0.00	0.08 ± 0.01	0.00 ± 0.00	***	***	***
18:1	19.92 ± 0.92	23.91 ± 1.30	17.48 ± 0.69	20.79 ± 0.77	***	***	n.s
20:1	1.50 ± 0.12	1.38 ± 0.25	1.64 ± 0.18	1.63 ± 0.21	*	***	n.s
22:1	0.04 ± 0.05	0.00 ± 0.00	0.03 ± 0.03	0.00 ± 0.00	***	n.s	n.s
Total monosatured FA	27.04 ± 0.95	28.18 ± 1.07	25.38 ± 1.02	25.17 ± 0.99	n.s	***	n.s
**Polyunsatured fatty acid (PUFA)**							
16:2 n-4	0.27 ± 0.05	0.00 ± 0.00	0.35 ± 0.03	0.14 ± 0.02	***	***	n.s
16:3 n-4	0.28 ± 0.02	0.10 ± 0.02	0.30 ± 0.02	0.02 ± 0.02	***	***	***
16:4 n-1	0.12 ± 0.02	0.00 ± 0.00	0.14 ± 0.02	0.05 ± 0.02	***	***	n.s
18:2 n-4	0.39 ± 0.02	0.06 ± 0.01	0.41 ± 0.02	0.00 ± 0.00	**	n.s	***
18:3 n-4	0.51 ± 0.02	0.06 ± 0.02	0.52 ± 0.04	0.03 ± 0.03	***	*	n.s
18:4 n-1	0.65 ± 0.04	0.09 ± 0.02	0.68 ± 0.08	0.00 ± 0.00	***	*	***
**PUFA n-6**							
18:2 n-6	5.14 ± 0.41	10.94 ± 1.27	4.09 ± 0.34	11.20 ± 0.71	***	**	***
18:2 n-4	0.39 ± 0.02	0.06 ± 0.01	0.41 ± 0.02	0.00 ± 0.00	**	n.s	***
18:3 n-6	0.16 ± 0.03	0.29 ± 0.06	0.06 ± 0.01	0.28 ± 0.06	***	n.s	***
20:2 n-6	1.04 ± 0.08	1.93 ± 0.24	0.95 ± 0.09	2.40 ± 0.32	***	**	***
20:3 n-6	0.60 ± 0.08	1.02 ± 0.07	0.55 ± 0.06	1.21 ± 0.17	***	**	***
20:4 n-6	1.80 ± 0.08	3.29 ± 0.20	1.74 ± 0.06	3.91 ± 0.22	***	***	*
22:2 n-6	0.08 ± 0.03	0.08 ± 0.04	0.15 ± 0.08	0.15 ± 0.04	***	***	*
22:4 n-6	0.10 ± 0.01	0.22 ± 0.04	0.12 ± 0.02	0.30 ± 0.05	***	***	***
22:5 n-6	0.15 ± 0.03	2.26 ± 0.22	0.15 ± 0.01	2.56 ± 0.19	***	***	***
Total PUFA n-6	11.29 ± 0.52	20.35 ± 1.05	10.23 ± 0.48	22.26 ± 0.85	***	n.s	***
**PUFA n-3**							
16:4 n-3	0.05 ± 0.02	0.00 ± 0.00	0.04 ± 0.02	0.07 ± 0.01	***	***	n.s
18:3 n-3	1.00 ± 0.09	3.03 ± 0.39	0.61 ± 0.09	2.90 ± 0.20	***	***	*
18:4 n-3	0.39 ± 0.06	0.39 ± 0.09	0.32 ± 0.04	0.35 ± 0.08	**	*	n.s
20:3 n-3	0.21 ± 0.05	0.40 ± 0.07	0.14 ± 0.02	0.40 ± 0.06	***	*	*
20:4 n-3	0.79 ± 0.10	0.54 ± 0.05	0.72 ± 0.11	0.41 ± 0.06	***	***	n.s
20:5 n-3	10.91 ± 0.54	5.07 ± 0.32	10.60 ± 0.71	3.58 ± 0.45	***	***	***
21:5 n-3	0.40 ± 0.01	0.07 ± 0.01	0.48 ± 0.04	0.00 ± 0.00	***	n.s	***
22:5 n-3	4.33 ± 0.33	1.77 ± 0.15	4.99 ± 0.62	1.25 ± 0.13	***	n.s	***
22:6 n-3	18.95 ± 1.45	20.09 ± 1.46	19.70 ± 1.36	21.40 ± 0.94	***	***	n.s
Total PUFA n-3	35.57 ± 1.38	27.94 ± 1.42	36.64 ± 0.89	27.04 ± 0.64	***	***	***
**Ratio**							
SFA/PUFA	0.50 ± 0.02	0.38 ± 0.03	0.53 ± 0.02	0.41 ± 0.02	***	***	n.s
n3 / n6	4.09 ± 0.31	1.57 ± 0.12	4.83 ± 0.25	1.38 ± 0.05	***	***	***
Index Insat	257.33 ± 6.52	253.43 ± 6.21	258.24 ± 3.97	253.14 ± 4.22	*	***	n.s
EPA/DHA	0.58 ± 0.06	0.25 ± 0.03	0.54 ± 0.07	0.17 ± 0.03	***	***	n.s
ARA/EPA	0.16 ± 0.01	0.65 ± 0.05	0.16 ± 0.01	1.11 ± 0.13	***	***	***
ARA/DHA	0.10 ± 0.01	0.16 ± 0.01	0.09 ± 0.01	0.18 ± 0.02	***	***	***

Values are means ± s.d. Two-way analysis of variance was carried out in order to assess effects of diet and cycle of reproduction. Replicates correspond to different individual egg batches (*n* = 10 per treatment). “*n.s*”: not significant; “*”: *P* < 0.05; “**”: *P* < 0.01; “***”: *P* < 0.001

Interestingly, although the proportion of EPA and DHA followed the same trend as in the diet (significantly more DHA and less EPA in eggs from MAB-fed females than in eggs from CB-fed females), the difference observed in the eggs was notably less important than in the diets. Eggs from MAB-fed females were enriched with EPA although the amount of EPA in the diet was very low. Eggs from CB-fed females, on the other hand, presented a higher proportion of DHA compared to that measured in the diet. Moreover, ARA, present in higher quantities in the CB diet compared to the MAB diet, was found, conversely, in higher quantities in eggs from MAB-fed females than those from CB-fed females. It should be noted that only a few differences were detected in the FA profile of eggs between Cycle 2 and Cycle 3.

### Progeny

#### Impact of the origin of the maternal diet on progeny performance

**Fig. 4:**
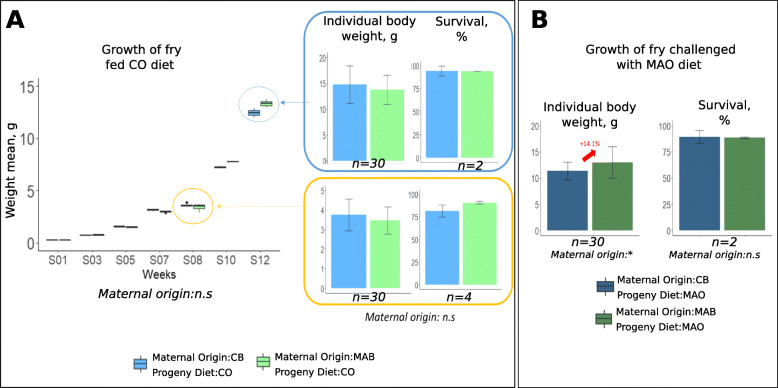
Effect of maternal nutrition on fry performances (**A**) during classic growth trial where fry were fed a CO diet for 12 weeks (evolution of mean body weight (g) during the 12 week-trial, individual weight (g) and survival percent (%) after 8 and 12 weeks) and (**B**) after 4 weeks of nutritional challenge where fry were fed the MAO diet (individual weight (g) and survival percent (%)). One-way analysis of variance was carried out in order to assess effects of maternal nutritional origin on individual final weight; replicates correspond to different individual fingerlings (*n* = 30 fingerlings per treatment). Kruskal-Wallis test was carried out to assess the effect of maternal nutritional origin on survival rate (*n* = 4 racks at week 8 and *n* = 2 racks at week 12) and the weight mean during rearing (*n* = 2 racks per treatment from week 0 to week 4 and *n* = 4 racks from week 5 to week 8). “n.s”: not significant; “*”: *P* < 0.05

During the growing period where fry were fed a commercial diet (CO; Fig. 4A), maternal origin did not affect the percent survival and the individual body weight of the fingerlings (*n* = 30 fingerlings per treatment) after 4 or 8 weeks of rearing (14.29 ± 3.15 g for progeny from CB-fed females versus 14.02 ± 2.55 g for progeny from MAB-fed females at the end of growth trial). The different measures of the mean weight carried out throughout the rearing period (once every 2 weeks) did not reveal any significant difference between the two maternal origins (*n* = 2 racks per treatment from week 0 to week 4 and *n* = 4 racks from week 5 to week 8).

After the nutritional challenge (i.e. progeny fed the MAO diet for 4 weeks), although no difference was observed on percent survival (90% for both maternal origins), the final individual weights of the fish (*n* = 30 fish per treatment) were significantly higher (*P* = 0.02) for progeny from MAB-fed females (12.98 ± 2.99 g vs. 11.65 ± 2.25 g) (Fig. 4B).

A biochemical analysis of the whole-body composition of the fingerlings (*n* = 4 pools of fingerlings per treatment) indicated no significant difference linked to the maternal diet in terms of lipids and proteins (Table 6).

**Table 6:** Proximate composition of progeny according to maternal nutritional origin and diet of progeny (fed a commercial diet or challenged with a MA diet)

Progeny diet	CO		MAO		Statistical results	
Maternal origin	CB	MAB	CB	MAB	Maternal origin	Progeny diet
Dry Matter, %	22.8 ± 0.3	21.9 ± 0.00	23.6 ± 0.3	23.9 ± 0.1	n.s	***
Protein, % WM	14.3 ± 0.5	13.81 ± 0.1	13.9 ± 0.2	13.8 ± 0.1	n.s	***
Lipids, % WM	7.1 ± 0.2	7.2 ± 0.2	9.1 ± 0.1	9.3 ± 0.2	n.s	***
Ash, % WM	2.0 ± 0.1	1.9 ± 0.1	1.7 ± 0.1	1.7 ± 0.1	n.s	***
Energy, kJ/g WM	5.8 ± 0.1	5.5 ± 0.3	6.4 ± 0.1	6.5 ± 0.1	n.s	**

Kruskal-Wallis test was carried out to assess the effects of diet and cycle of reproduction on proximate composition. Replicates correspond to different pools of fingerlings (*n* = 4 per treatment). Values are means ± s.d. “*n.s*”: not significate; “*”: *P* < 0.05; “**”: *P* < 0.01; “***”: *P* < 0.001. *WM* = wet matter.

#### Impact of the origin of the maternal diet on the lipid metabolism of the progeny

**Fig. 5:**
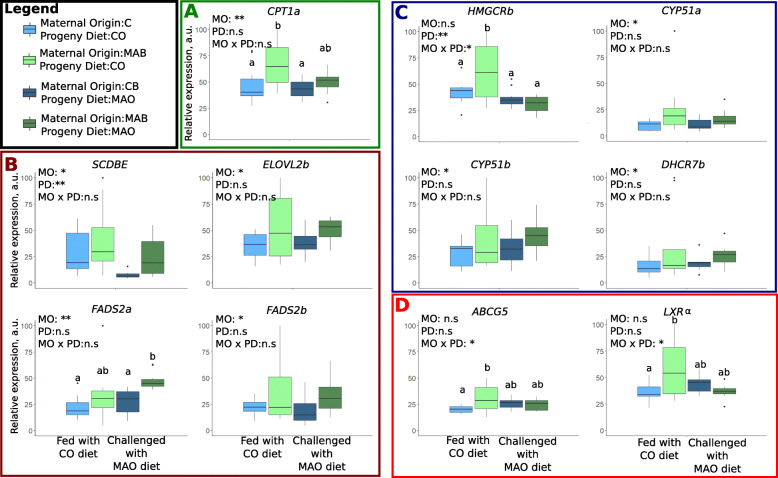
Relative expression of genes involved in hepatic lipid metabolism of the progeny according maternal origin (MO) and progeny diet (PD: fed a C diet or challenged with a MA diet). Only genes where a significant effect of maternal origin are represented. Other results are presented in Additional file 3: Table S3. Different pathways of pentose phosphate (**A**), of β-oxidation FA (**B**), of biosynthesis of PUFA (**C**), of cholesterol synthesis (**D**) and of cholesterol elimination (**E**) are represented in different boxes. Two-way analysis of variance was carried out in order to assess effects of maternal nutritional origin and diet of progeny. Replicates correspond to liver from different individual fingerlings (*n* = 10 per treatment). “n.s”: not significate; “*”: *P* < 0.05; “**”: *P* < 0.01; “***”: *P* < 0.001. a.u = arbitrary unit. Different letters indicate significant differences between groups, which were investigated with a Tukey post hoc test

Maternal nutrition affected lipid metabolism of the progeny that were fed the CO diet or challenged with the MAO diet (*n* = 10 livers from individual fingerlings per treatment) (Fig. 5). The FA β-oxidation pathway of progeny seems to be affected by maternal nutrition origin. The expression of the hepatic *CPT1a* gene was up-regulated in progeny from MAB-fed females (Fig. 5A), regardless of the diet of the offspring. Further, the CO diet magnified the impact of the maternal nutritional history on the hepatic expression of *CPT1a*.

Similarly, feeding female broodstock the MAB diet led to an up-regulation of some genes involved in LC-PUFA biosynthesis in the progeny. Thus, offspring exhibited an up-regulation of hepatic *SCDB*, *ELOVL2b, FADS2a* and *FADS2b* when female broodstock were fed the MAB diet (Fig. 5B). The nutritional challenge with MAO diet accentuated the difference of *FADS2a* gene expression between the progeny from MAB-fed females and those from CB-fed females.

Finally, the maternal nutritional history also affected cholesterol biosynthesis in the liver. Higher relative expression of *CYP51a* and *b*, and *DHCR7* were recorded in the progenies of MAB-fed females, whether fed with a CO diet or challenged with a MAO diet. A different expression pattern was observed for *HMGCRb* which was up-regulated in fry from MAB-fed females but only when fed the CO diet (Fig. 5C).

Genes involved in the elimination of cholesterol were only affected by maternal nutritional history when progeny were fed the CO diet as illustrated by the up-regulation of *ABCG5* and *LXRα* gene expression in the fry from MAB-fed females (Fig. 5D).

## Discussion

FM and FO replacement in aquaculture diet is the key issue for maintaining sustainable aquaculture production. Although research on this topic for growing trout is well advanced, there is little research on the impact of this replacement on broodstock and their offspring. The first objective of the present study was to determine if microalgae could be an efficient alternative source of DHA in a plant-based diet dedicated to female broodstock. The second objective was to investigate the possible implementation of a nutritional programming in the offspring, related to maternal nutritional history.

However, we would advise that in the present study, we have chosen to highlight the impact of the *Schizochytrium* sp. supplementation in plant diet on lipid metabolism due to the high DHA content of this micro-algae and of the important role of this fatty acid in the trout reproduction but it was not possible to reject completely any effect on the effect on reproductive performance and progeny of other factors such as dietary protein content, amino acid profile additives, etc. 

### Validation of DHA-rich microalgae as supplementation in a plant-based diet for female broodstock

The present study indicates that for rainbow trout female broodstock, a plant-based diet containing *Schizochytrium* sp. micro-algae as an alternative source of DHA could be as efficient as the current commercial diet, during the reproductive cycle. Despite a significantly lower growth rate for females fed the plant-based diet containing *Schizochytrium* sp. micro-algae, the reproductive performances, namely egg weight, absolute fecundity and relative fecundity, were similar to broodstock females fed a commercial diet containing fishmeal and fish oil. Moreover, during the second reproductive cycle, even if egg diameter was lower, eggs produced by females fed the plant-based diet supplemented with microalgae exhibited a greater integrity illustrated by fewer white eggs after 24 h of hydration, which indicates a better overall egg quality. The presence of white eggs can be interpreted as an indirect measure of vitelline membrane integrity. Therefore, when eggs are of good quality, the vitelline membrane is highly impermeable to water and electrolytes which reduces the risk of rupture of the membrane [36]. Inversely, when eggs are of bad quality, the vitelline membrane is weakened and the water pressure is enough to break it. In this case, eggs turns white during hydration [35]. The higher fry percent survival after resorption also confirmed higher egg quality from females fed the plant-based diet. In a previous study, Lazzarotto et al. [3] showed that a plant-based diet completely devoid of long chain PUFAs had negative consequences on rainbow trout reproductive performances and egg quality by reducing the egg size and the survival of the offspring. As reproductive performance was not impaired in the present study, it can be assumed that the addition of DHA-rich microalgae can limit the negative effects of plant-based diets on reproduction.

### Possible improvement of egg quality through a fatty acid profile that appears more suitable with a DHA-rich microalgae plant diet

The vitellus reserves are intended to feed the embryo endotrophically until the complete resorption of the yolk sac and the transition to exotrophic feeding [40]. In the present study, the difference in FA profile of the eggs could explain the higher quality of eggs produced by females fed the plant-based diet. The FA composition of egg reserves is a good proxy for the female broodstock diet, further supporting results reported in previous studies on rainbow trout [3] or other fish species [13, 40]. However, although the diets used in this study contained very distinct levels of DHA, the differences observed in the eggs were considerably less significant than in the diets. This result confirms that DHA is selectively accumulated in eggs regardless of its level in the diet [40, 41]. A significant proportion of EPA was found in the eggs of females fed the commercial diet (10% to 12%), reflecting the high EPA content of the commercial diet. Further, despite the near absence of EPA in the plant-based diet, EPA was also detected in eggs from females fed the plant-based diet, in non-negligible proportions (between 3.9% and 5.1%). Two hypotheses can be proposed to explain this phenomenon. As during the first breeding eggs are of poor quality and barely used for fry production, the experiment started with broodstock females, just after their first reproduction. During this period, fish were fed the standard commercial diet. It is known that the n-3 PUFAs are stored in the female’s tissue reserves for further use during development and then selectively mobilized and incorporated into the eggs during vitellogenesis [42]. It is thus possible that the EPA present in the eggs of females fed the experimental plant-based diet, which was almost devoid of EPA, comes from the tissue reserves accumulated by the female before the first reproduction and then mobilized for the benefit of the eggs during the second and third reproductive periods. However, EPA could have also been biosynthesized from its precursor, the ALA, present in large quantities in the plant-based diet [43, 44]. The two phenomena, the mobilization of reserves or biosynthesis, can proceed at the same time.

Finally, the concentrations of ARA were also of interest. In the eggs produced by the females fed the plant-based diet supplemented with microalgae, the proportion of ARA was significantly higher than in females fed the control diet, though it was much less abundant in the plant-based diet (0.2% vs. 0.8%). This demonstrates a selective incorporation of ARA into the eggs of females fed the plant-based diet. Thus, ARA was probably biosynthesized from linoleic acid (LA) and stored in the eggs of the females fed the plant-based diet enriched with DHA. This increased proportion of ARA and the higher ARA/EPA ratio in the eggs of females fed the plant-based diet might explain the higher fry survival after resorption. In fish, ARA plays a major role in reproduction [45–47] and egg quality [40, 48, 49]. In both mammals and fish, ARA and EPA are considered as the precursors of eicosanoids, namely prostaglandin (Pg), thromboxane (Tx) and leukotriene (Lt) but ARA is considered the most active eicosanoid precursor in fish [48]. The competitive relationship between ARA and EPA in the production of eicosanoids has drawn attention to the importance of the EPA/ARA ratio in addition to the individual levels of these PUFAs [15]. Furthermore, EPA and ARA compete for the same metabolic and enzymatic pathways [40] which could explain the difference in egg quality observed in this study. Thus, the present study suggests that the MAB diet had a more suitable profile of PUFAs which could result in better egg quality. Results from this study confirm the importance of the balance between PUFAs and highlight a specific requirement for ARA in female rainbow trout broodstock. Further studies are necessary to determine the specifics of the requirement in rainbow trout.

### Confirmation of nutritional programming through maternal nutrition

The vitellus reserves of the egg are known to affect progeny survival and development and are at the origin of early nutritional programming of phenotypes in fish [13]. Nutritional programming is emerging as a potential strategy to modulate some metabolic pathways and to improve the ability of fish to grow despite being fed a challenging diet. This study supports this assumption. Indeed, the present results demonstrate that the utilization of plant-based diets in rainbow trout could be improved by maternal nutrition. Feeding females a plant-based diet supplemented with micro-algae improves growth in 4-month-old progeny challenged with a plant-based diet also supplemented with microalgae. This is in accordance with previous results on gilthead sea bream which showed that replacement of FO with linseed oil in broodstock diets improved growth in 4-month-old progeny challenged with low FO and FM diets for a period of 1 month [13].

Nevertheless, the effect of programming was revealed regardless of the diet fed to the progeny. At the molecular level, whether the progeny is fed a commercial diet or challenged with a plant-based diet supplemented with DHA, lipid and cholesterol metabolisms were affected by the maternal nutrition. The capacity of endogenous biosynthesis of cholesterol and LC-PUFAs were enhanced in the progeny of females fed the plant-based diet, at the messenger RNA level.

The higher expression of genes involved in cholesterol synthesis (*HMGCRb*, *CYP51a*, *CYP51b*, *DHCR7b*) could be a consequence of the near absence of cholesterol in the maternal diet. This maternal cholesterol deficiency, which induces a significant decrease in the plasma cholesterol level in female broodstock, could be at the origin of a parental nutritional programming of the offspring leading to an increased capacity of the progeny to biosynthesize cholesterol in anticipation of a possible absence of cholesterol in the diet. Maternal cholesterolemia is known to affect the hepatic cholesterol metabolism in mouse offspring [50]. This kind of nutritional programming through maternal nutrition thus seems to trigger a “predictive adaptive response” to quote the words of Gluckman et al. [51]. Increased expression of genes involved in the cholesterol elimination pathway (*ABCG5* and *LXR*) in progeny from females fed a plant-based diet is only observed when the progeny was fed the commercial diet. The neosynthesized cholesterol added to the cholesterol supplied by the commercial diet likely led to a high accumulation of cholesterol, hence an increase of its elimination only when the progeny was fed with a diet rich in cholesterol.

Carnitine palmitoyltransferase-1 is considered as a rate-limiting step of the mitochondrial β-oxidation by controlling the mitochondrial uptake of long-chain acyl-CoAs. Expression of *CPT1* in the liver of rainbow trout is strongly, directly and differently regulated by individual FAs. EPA down-regulates *CPT1* expression in rainbow trout while DHA has the opposite effect [52]. The different contents of EPA and DHA between the commercial diet and the DHA enriched plant-based diet given to fingerlings did not lead to a differential expression of *CPT1* in the progeny. However, the MAB diet seems to be responsible for a higher expression of *CPT1* in the progeny. This highlights the occurrence of another maternal nutritional programming process, which could be linked to the FA profile of the maternal diet.

Maternal nutrition also induced changes in LC-PUFA biogenesis. Progeny of females fed the plant-based diet supplemented with microalgae exhibited higher expression of genes involved in elongation and desaturation of n-3 and n-6 FAs. Such changes are consistent with previous results showing that feeding gilthead sea bream broodstock different FA profiles affects the LC-PUFA biosynthesis pathway of the progeny [13, 53]. Long-chain PUFA can be synthesized from ALA and LA in vertebrates through a series of desaturation and elongation reactions [54]. Therefore, the higher expression of *SCBE*, *FADS2a*, *FADS2b* and *ELOVL2b* could be an adaptation to the very high proportion of ALA and LA supplied by the MAB broodstock diet [13].

## Conclusion

To conclude, the present study demonstrates that supplementation of a plant-based diet with DHA-rich microalgae yields reproductive performance and egg quality comparable to those observed with a conventional commercial feed containing FM and FO. Moreover, we show that feeding a plant-based diet enriched in DHA to rainbow trout broodstock females could induce metabolic programming processes in the progeny that may be related to the lipid profile of the maternal diet. In order to better understand these programming events, the underlying molecular mechanisms will need to be characterized. Particular attention should be paid to epigenetic mechanisms known to orchestrate programming by maternal nutrition [55].

## Supplementary Information

**Additional file 1: Table S1.** Main fatty acid (% of total FA) of progeny diets (C and MA).

**Additional file 2: Table S2.** Sequences of primer pairs used for gene expression analysis by qRT-PCR.

**Additional file 3: Table S3.** Relative expression of genes involved in lipid metabolism of the progeny according maternal origin (MO) and progeny diet (PD: fed a C diet or challenged with a MA diet). Two-way analysis of variance was carried out in order to assess effects of maternal nutritional origin and progeny diet. Replicates correspond to liver from different individual fingerlings (*n* = 10 per treatment). Values are means± s.d. “n.s”: not significative; “*”:*P* < 0.05; “**”:*P* < 0.01; “***”:*P* < 0.001. a.u = arbitrary unit.

## Data Availability

All data generated or analyzed during this study are available from the corresponding author on reasonable request.

## Abbreviations

FM: Fishmeal
FO: Fish oil
SFA: Saturated fatty acid
MUFA: Monounsaturated fatty acid
LC-PUFA: Long chain polyunsaturated fatty acid
EPA: Eicosapentaenoic acid (20:5 n-3)
DHA: Docosahexaenoic acid (22:6 n-3)
LA: Linoleic acid (18:2 n-6)
ALA: Linolenic acid (18:3 n-3)
DPA: Docosapentaenoic acid (22:5 n-6)
ARA: Arachidonic Acid (20:4 n-6)
ACLY: ATP cytrate lyase
FAS: Fatty acid synthase
CPT1a: Carnitine palmitoyl transferase 1
HADH: HydroxyacylcoA dehydrogenase
ACOX: AcylcoA oxidase
SCDB: Acyl-CoA desaturase - delta-9 desaturase
ELOVL2: Elongation of very long chain fatty acids protein 2
ELOVL5: Elongation of very long chain fatty acids protein 5
FADS2: Fatty acid desaturase 2
HMGCS: 3-hydroxy-3-methylglutaryl-CoA synthase 1
HMGCR: 3-hydroxy-3-methylglutaryl-CoA reductase
CYP7a: Cholesterol 7 alpha hydroxylase a
CYP51: Lanosterol 14-alpha demethylase
ABCA1: ATP-binding cassette sub-family A member 1-like
ABCG5: ATP binding cassette subfamily G member 5
ABCG8: ATP binding cassette subfamily G member 8
DHCR7: 7-dehydrocholesterol reductase
